# Clinical features and antinuclear antibodies profile among adults with systemic lupus erythematosus and lupus nephritis: a cross-sectional study

**DOI:** 10.11604/pamj.2017.27.114.5981

**Published:** 2017-06-14

**Authors:** Nashwa Ahmed, Mazin Shigidi, Al Nour Al Agib, Hassan Abdelrahman, Elshafie Taha

**Affiliations:** 1 Sudan Medical Specialization Board, Ministry of Health, Khartoum, Sudan; 2 Salma Centre for Kidney Diseases, Faculty of Medicine, University of Khartoum, Sudan; 3 Faculty of Medicine, Karrai University, Sudan; 4 Faculty of Medicine, University of Medical Sciences and Technology, Sudan; 5 Cardiology Department, Al Ain Hospital, United Arab Emirates

**Keywords:** SLE, lupus nephritis, ANA profile, Sudan

## Abstract

**Introduction:**

Limited data is available regarding the clinical manifestations and pattern of Systemic Lupus Erythematosus (SLE) in Sudan. This study aimed to determine the clinical manifestations and Antinuclear Antibodies (ANA) profile among Sudanese adults with SLE and lupus nephritis (LN).

**Methods:**

A descriptive study was conducted in Omdurman Military Hospital, Sudan. It included all adults with SLE and on regular follow-up during the study period (December 2012 to May 2013). These were investigated regarding their demographic details, clinical features, and immunological profile (ANA, anti-double stranded DNA, and ANA profile 3 levels). Patients with LN had their pattern of renal involvement described; furthermore, associations between the various SLE reactive antibodies and the histological diagnosis of lupus were studied.

**Results:**

Sixty-two Sudanese adults with SLE were included, their mean age was 31 ± 10.9 year. Females made 93.5% of patients. A clear predominance of those of Arab ancestry was seen, with most patients being from the Ja'alin and Shaigiya ethnic groups accounting for 29% and 12.9%, respectively. Arthritis was the dominant clinical manifestation seen in 85.5%, whereas renal involvement was seen in 66.1% of patients. Lupus nephritis class III was the dominant histological lesion, seen in 39% of patients. On correlating the ANA profile to the histopathological diagnosis of LN, anti-Nucleosomes and anti-AMA-M2 autoantibodies were found to be significantly associated with LN class IV and class VI, respectively (P values < 0.05).

**Conclusion:**

Further epidemiological studies regarding SLE and its ANA profile remain essential as they might help predicting the clinical patterns of the disease and its prognosis.

## Introduction

Systemic lupus Erythematosus (SLE) is a multisystem chronic inflammatory autoimmune disease characterized by periods of remissions and relapses. The reported prevalence of SLE in the population is 20 to 150 cases per 100,000 [1–3]. Due to improved detection of mild disease, the incidence of the disease has almost tripled over the last 40 years [4, 5]. The disease appears to be rare in west Africa, but showing an increasing frequency in the central and southern parts of the continent [6]. In Sudan, limited data is available regarding the incidence and prevalence of SLE, though a clear ethnic distribution of the disease was previously reported, with the disease being mostly prevalent among Sudanese Arabs with Nubian (Kushite) ancestry compared to those of central African descent [7, 8]. Lupus Nephritis was reported to account for 11.4%-14.7% of all cases with glomerular diseases, who had kidney biopsy [8, 9]. This study aimed to investigate the clinical presentation of SLE and LN among Sudanese adults, determine the Antinuclear antibodies (ANA) profile and look for probable associations between the ANA pattern and the clinical manifestations of LN.

## Methods

A descriptive hospital-based cross-sectional study was conducted in Omdurman Military Hospital, Khartoum State, Sudan. Selection of the study area was based on the presence of an Internal Medicine department with a highly specialized nephrology and rheumatology units, as well as the presence of accessible Hospital medical records system for research purposes. Enrolled patients were those previously diagnosed as having SLE and receiving regular follow-up in the specified hospital during the period from December 2012 to May 2013. The Systemic Lupus International Collaborating Clinics (SLICC) group revised and validated American College of Rheumatology (ACR) SLE classification 2012 criteria was applied for the diagnosis of SLE in our study [10, 11]. Accordingly, patients were diagnosed as having SLE if they satisfied four of the ACR diagnostic criteria including at least one clinical and one immunologic criterion, or had biopsy-proven lupus nephritis with ANA or anti-double stranded DNA (anti-dsDNA) antibodies [11]. Exclusion criteria were; age less than 18 years, patients with incomplete medical records, patients with kidney diseases and not fulfilling the diagnostic criteria of lupus, patients with long standing diabetes and /or hypertension together with target organ damage, patients with reactive Hepatitis B, Hepatitis C and /or HIV on screening, and those who refused or were unable to give consent for enrollment. Information regarding patients' demographic details at diagnosis (age, sex, ethnic group, marital status) and clinical manifestations were collected. Laboratory investigations including Antinuclear antibodies (ANA) profiles were measured using immunoblot technique.

Further serological information obtained included autoantibodies against protein autoantigens including nRNP/Sm, Sm, Ro52, ss-B, Scl 70, PM-Scl, Jo-1, CENP-B, dsDNA, nucleosomes, PCNA, Histones, Rib.P-protien and AMA-M2, La, and topoisomerase I (Scl-70) [12, 13]. Patients with LN had their clinical data compared with that of those having SLE but no evidence of renal involvement. Clinical absence of renal involvement was based on the absence of hematuria, proteinuria, and cellular casts in prior urine analysis and 24-hour urine collection for protein, together with a normal serum creatinine and estimated glomerular filtration rate. [12, 14]. Associations between the various SLE reactive antibodies and the histological classification of lupus were targeted. All patients with lupus nephritis had ultrasound guided kidney biopsy for disease classification as per the International Society of Nephrology/Renal Pathology Society ISN/RPS 2003 classification of lupus [15]. Obtained data was entered into a specifically designed questionnaire and analyzed using Statistical Package for the Social Sciences (SPSS) Version 20.0. Descriptive analysis of data was done and results were expressed as percentages and means or medians with standard deviation. Comparative analysis of variables was done using Pearson's Correlation Coefficient to assess possible relationships between lupus nephritis and the ANA profile. Multiple Linear regressions were applied to determine the explanation of the different variables to the Larsen score. The study was approved by the ethical committee of the Sudan Medical Specialization Board.

## Results

Sixty-two adult Sudanese patients with SLE were studied, their mean age was 31 ± 10.9 years (range 18-62 years); with a mean age at diagnosis of lupus of 27 ± 10.2 years. Females made 93.5% of patients. Most of the study population, 62.9%, were residing in the capital city Khartoum; whereas 37.1% were from rural areas. A clear predominance of those of Arab ancestry was seen, with most patients being from the Ja'alin, Shaigiya, Brno, Gmoea and Mahas ethnic groups accounting for 29%, 12.9%, 4.8%, 4.8% and 4.8% of patients, respectively. Among the study population 27.4% gave a history of previous episodes of relapses and remissions. Morbid pregnancies were reported in 31 females, 25.8% of all married females. As per the ACR criteria for the diagnosis of lupus, arthritis was the most predominant clinical feature seen in 85.5% of patients, followed by renal and hematological manifestations in 66.1% and 53.2% of patients, respectively (Table 1). The ANA profile showed a positive anti-dsDNA in 48.4% of patients, anti-RO 52 antibodies in 41.9% and anti-SSm in 32.3%. Anti Scl 70 antibodies were not detected in any of the study population (Table 2). Forty-one patients (66.1%) were known to have SLE with evident LN. Their ages ranged between 18 - 62 years, with a mean age at diagnosis of SLE of 24 ± 7.4 years. Among these a family history of SLE was prevalent in 2 patients (4.9%), prior thrombotic events were diagnosed in 2 patients (4.9%) and morbid pregnancies were reported in 3 females, 7.5% of all married females.

**Table 1:** Demographic features and ACR diagnostic criteria among patients with SLE (N = 62)

Prevalent features	Frequency (%)
Demography	
Female: Male ratio	58 (93.5%) / 4 (6.5%)
Mean age of study population	31 ± 10.9 years
Mean age at diagnosis of SLE	27 ± 10.2 years
Family history of SLE	9 (14.5%)
Prior thrombotic events	9 (14.5%)
* ACR criteria	
Hair Loss	20 (32.3%)
Malar rash	27 (43.5%)
Discoid rash	17 (27.4%)
Photosensitive rash	19 (30.6%)
Oral ulcer	19 (30.6%)
Arthritis	53 (85.5%)
Serositis	10 (16.1%)
Hematological	33 (53.2%)
Neurologic	22 (35.5%)
Renal	41 (66.1%)

* American College of Rheumatology

**Table 2:** ANA profile positivity among Sudanese adults with SLE

ANA profile	Frequency (%)
Anti dsDNA antibodies	30 (48.4%)
Anti RO52 antibodies	26 (41.9%)
Anti SS-A antibodies	20 (32.3%)
Anti Nucleosomes antibodies	14 (22.6%)
Anti Sm antibodies	12 (19.3%)
Anti RNP/Sm antibodies	11 (17.7%)
Anti Histones antibodies	10 (16.1%)
Anti PCNA antibodies	7 (11.3%)
Anti SS-B antibodies	7 (11.3%)
Anti Ribosomal-P-protein antibodies	4 (6.5%)
Anti JO1 antibodies	3 (4.8%)
Anti AMA-M2 antibodies	3 (4.8%)
Anti Centromere B antibodies	1 (1.6%)
Anti PM-Scl antibodies	1 (1.6%)

Thirteen patients (31.7%) gave a history of prior episodes of relapses and remissions. When compared to those with SLE and no evidence of renal involvement, patients with LN tend to have their disease onset at a relatively younger age and showed lesser incident of SLE among their family members, with P values of less than 0.05 (Table 3). Arthritis was the most predominant clinical feature seen in 34 patients (82.9%); whereas hematological manifestations, malar rash, discoid rash, oral ulcers, and neurological disorders were seen in 58.5%, 46.3%, 29.3%, 29.3% and 26.8% of patients with LN, respectively. Hair loss and neurological manifestations were observed less frequently among patients with LN when compared to those with SLE and no evidence of renal involvement, with P values of less than 0.05 (Table 4). The Antinuclear Antibody (ANA) profile for all patients was done using the immunoblot technique. None of our SLE patients had detectable Anti Scl 70 antibodies in their serum. Patients with LN had no detectable anti JO1 and Centromere B antibodies; whereas significantly less prevalent antibodies against JO1 and PCNA were seen in those with LN, P values of less than 0.05 (Table 5). Histological analysis of renal biopsies revealed that lupus nephritis class III was the dominant histological finding, seen in 39% of patients; that was followed by LN class IV, II, VI and V seen in 29.3%, 22%, 7.3% and 2.4% of patients, respectively. On correlating the ANA profile to the histopathological diagnosis of LN, anti-Nucleosomes and anti-AMA-M2 antibodies were found to be significantly associated with LN class IV and VI, respectively (Table 6).

**Table 3:** Demographic features of Sudanese adults with lupus nephritis

Demographic features	SLE patients		P value
	with lupus nephritis(N=41)	without renal involvement (N=21)	
Mean age at disease onset	24 ± 7.4	33 ± 12.6	0.008*
Male: Female ratio	1 (2.4%) / 40 (97.6%)	3 (14.3%) / 18 (85.7%)	0.1
Family history of SLE	2 (4.9%)	7 (33.3%)	0.002*
Morbid pregnancy	3 (7.3%)	3 (14.3%)	0.052
Thrombotic events	2 (4.9%)	3 (14.3%)	0.2
Prior SLE relapses	13 (31.7%)	4 (19%)	0.26

**Table 4:** ACR diagnostic criteria among Sudanese adults with lupus nephritis

ACR diagnostic criteria	ACR criteria in SLE patients		P value
	with lupus nephritis (N=41)	without renal involvement (N=21)	
Hair Loss	9 (21.9%)	11 (52.4%)	0.015*
Malar rash	19 (46.3%)	8 (38.1%)	0.55
Discoid rash	12 (29.3%)	5 (23.8%)	0.65
Photosensitive rash	10 (24.4%)	9 (42.9%)	0.14
Oral ulcer	12 (29.3%)	7 (33.3%)	0.74
Arthritis	34 (82.9%)	19 (90.5%)	0.42
Serositis	6 (14.6%)	4 (19%)	0.65
Hematological	24 (58.5%)	9 (42.9%)	0.24
Neurological	11 (26.8%)	11 (52.4%)	0.047*

**Table 5:** ANA profile positivity among Sudanese adults with lupus nephritis

ANA profile	SLE Patients		P value
	with lupus nephritis (N=41)	without renal involvement (N=21)	
Anti RNP/Sm antibodies	8 (19.5%)	3 (14.3%)	0.61
Anti Sm antibodies	7 (17.1%)	5 (23.8%)	0.52
Anti SS-A antibodies	10 (24.4%)	10 (47.6%)	0.06
Anti RO52 antibodies	16 (39%)	10 (47.6%)	0.51
Anti SS-B antibodies	3 (7.3%)	4 (19%)	0.16
Anti PM-Scl antibodies	1 (2.4%)	0 (0%)	0.47
Anti JO1 antibodies	0 (0%)	3 (14.3%)	0.01*
Anti Centromere B antibodies	0 (0%)	1 (4.8%)	0.16
Anti PCNA antibodies	2 (4.9%)	5 (23.8%)	0.02*
Anti dsDNA antibodies	22 (53.7%)	8 (38.1%)	0.25
Anti Nucleosomes antibodies	11 (26.8%)	3 (14.3%)	0.26
Anti Histones antibodies	7 (17.1%)	3 (14.3%)	0.78
Anti ^†^ RP-protein antibodies	2 (4.9%)	2 (9.5%)	0.48
Anti AMA-M2 antibodies	1 (2.4%)	2 (9.5%)	0.22

† Ribosomal-P-Protein

**Table 6:** ANA profile distribution along the various histological classes of lupus nephritis

ANA profile	Histological classification of lupus nephritis					P value
	Class II	Class III	Class IV	Class V	Class VI	
	(N = 9)	(N = 16)	(N = 12)	(N = 1)	(N = 3)	
Anti RNP/Sm antibodies	1 (11.1%)	2 (12.5%)	5 (41.7%)	0 (0%)	0 (0%)	0.23
Anti Sm antibodies	1 (11.1%)	1 (6.25%)	5 (41.7%)	0 (0%)	0 (0%)	0.11
Anti SS-A antibodies	1 (11.14%)	6 (37.5%)	2 (16.7%)	0 (0%)	1 (33.3%)	0.53
Anti RO52 antibodies	2 (22.2%)	8 (50%)	5 (41.7%)	0 (0%)	1 (3.3%)	0.63
Anti SS-B antibodies	0 (0%)	3 (18.75%)	0 (0%)	0 (0%)	0 (0%)	0.28
Anti PM-Scl antibodies	0 (0%)	0 (0%)	1 (8.3%)	0 (0%)	0 (0%)	0.65
Anti PCNA antibodies	1 (11.1%)	0 (0%)	1 (8.3%)	0 (0%)	0 (0%)	0.72
Anti dsDNA antibodies	5 (55.6%)	7 (43.75%)	9 (75%)	1 (100%)	0 (0%)	0.13
Anti-Nucleosomes antibodies	0 (0%)	3 (18.75%)	8 (66.7%)	0 (0%)	0 (0%)	0.005*
Anti-Histones antibodies	0 (0%)	2 (12.5%)	4 (33.3%)	0 (0%)	1 (33.3%)	0.29
Anti^†^ RP-protein antibodies	0 (0%)	2 (12.5%)	0 (0%)	0 (0%)	0 (0%)	0.51
Anti AMA-M2 antibodies	0 (0%)	0 (0%)	0 (0%)	0 (0%)	1 (33.3%)	0.011*

† Ribosomal-P-Protein.  No patients with lupus nephritis had a positive anti** **Scl-70, JO1 or Centromere B antibodies

## Discussion

Systemic Lupus Erythematosus had been repeatedly described with significant geographic and racial variations [16–18]. The disease is mostly seen in females, with the younger women being mostly affected [19]. In our study, the female to male ratio was 14.5:1, a figure close to that reported in a study from California and Pennsylvania [2]. The increased frequency of SLE among women had been attributed in part to a postulated estrogen hormonal effect [8, 20]. Most of our study patients were from urban areas, being the disease mostly prevalent in urban rather than rural areas [2, 5]. Again, a clear tribal preponderance was evident with most of our patients being from the Ja´alia and Shaigiya ethnic background, these are predominantly Arab tribes with Nubian ancestry. A previous report from Sudan showed a similar ethnic variation, with the disease being less frequently seen among Nilotic and Western Sudanese tribes [8]. SLE is a multisystem disease with the skin and joints being predominantly affected [21]. Among our patients, arthritis was the most common clinical manifestation reported in 85.5%; a figure close to that published in various previous reports. Similarly, constitutional symptoms in the form of fever, fatigability and weight loss were prevalent in 72.6% of our patients; these had often been previously reported in 50% to 100% of lupus patients [22–25]. Neurological manifestations were seen in 35.5% of our patients, mostly in the form of persistent unexplained headache; whereas serositis was seen in 16.1% of patients predominantly in the form of pleural effusions. Hematological abnormalities had been reported in 40% of SLE patients, mostly in the form of leucopenia, autoimmune hemolytic anemia, and thrombocytopenia in 25%, 14% and 7% of patients, respectively [26, 27]. Among the study group hematological manifestations were seen in 53.2% of patients, often in the form of unexplained anaemia. In the literature, renal involvement was reported to occur in 50% of SLE cases though subclinical diseases can occur. Lupus nephritis usually manifest early in the first few years of the disease, often detected by periodic urine testing and estimation of the glomerular filtration rate. Kidney biopsy remains essential to define the type and extent of renal involvement [26–28]. We compared the clinical features in patients with SLE and LN to those with no evidence of nephritis. Hair Loss, the presence of neurological manifestations and a family history of connective tissue disease were significantly seen in those with no evidence of renal involvement (P value < 0.05).

An elevated ANA titer remains one of the diagnostic criteria for SLE even though it has low specificity and can occur in many other autoimmune diseases, some infectious diseases, malignancies and may be drug induced. In contrast to ANA individual autoantigen antibodies tend to be more sensitive and specific [10, 29, 30]. Several studies had demonstrated that specific ANA are associated with different clinical manifestations of SLE. [26, 31–33] No prior data is available comparing ANA profile in patients with LN to those with SLE and no evidence of renal involvement. Considering the ANA profile of the two groups, anti-ds-DNA antibodies were more prevalent in patients with LN, thought that was not statistically significant. On the other hand, anti JO1 antibodies were not detected in patients with LN, but seen in 14.3% of those with no evidence of kidney disease; a finding which was statistically significant with a P value of 0.013. Furthermore, anti Nucleosome antibodies despite being strongly correlated to LN did not seem to be significantly prevalent among our patients with LN. Anti Histones antibodies were detected almost equally in patients with and without nephritis; the presence of this antibody is known to be strongly associated only with drug induced SLE and remains a predictor of disease remission with withdrawal of the offending drug [34, 35]. Scl-70 antibodies were not detected in any of our patients, this antibody is known to be associated with Centromere B which was prevalent in 4.76% of our SLE patients with no evidence of nephritis, the antigen is reported to be predominantly present during cell division [35, 36]. PCNA was detected in 23.8% of SLE patients without Nephritis compared to 4.9% of patients with LN patients, a statistically significant variability with a P value of 0.026. Among our patients LN class III was the dominant histological pattern seen in 39% of patients with nephritis, a finding like that previously reported from Saudi Arabia [37], and Kuwait [38]. None of our patients had LN class I on histopathology. The ANA profile is known to provide a higher sensitivity for identifying patients with autoimmune disease compared to the ANA test alone [39]. We found a statistically significant association between detected auto-antibodies and the histological diagnosis of LN. Anti-Nucleosomes antibody was detected more frequently in patients with LN class IV the most severe form of the disease (P value < 0.005). On the other hand, AMA-M2 were detected only in LN class VI (P value 0.011) and seems to be associated with sclerotic changes in the renal parenchyma. Limitations of this study include the retrospective nature of its design in addition to the small sample size studied.

## Conclusions

Patients with SLE may present with variable combinations of clinical features and serologic evidence of lupus [11]. Among the Sudanese patients the Ja´alia and Shaigiya were the most commonly affected ethnic groups. Arthritis remains the commonest clinical manifestation followed by constitutional symptoms and renal involvement. Using an immunoblot technique striking associations between specific autoantibodies, and LN class IV and VI were detected. Accordingly, patients´ ANA profile might provide additional diagnostic and prognostic information about LN based on the autoantigens detected [40]. Further epidemiological studies about SLE and its ANA profile remains essential among Sudanese patients; these might help in predicting the clinical patterns of the disease and its prognosis.

### What is known about this topic

- Lupus nephritis shows a clear ethnic and tribal distribution;
- Arthritis remains the most common clinical manifestation of SLE;
- In Sudan, LN was diagnosed in 11.4% to 14.7% of patients with glomerulonephritis who had a kidney biopsy.

### What this study adds

- Patients with SLE and nephritis tend to have their disease onset at a relatively younger age;
- Anti-dsDNA antibodies were more prevalent among SLE patients with evidence of nephritis;
- The pattern of auto-antibodies detected correlated significantly with the histological classification of LN.

## Competing interests

The authors declare no competing interests.
